# Acute Fluoxetine Treatment Induces Slow Rolling of Leukocytes on Endothelium in Mice

**DOI:** 10.1371/journal.pone.0088316

**Published:** 2014-02-10

**Authors:** Nadine Herr, Maximilian Mauler, Thilo Witsch, Daniela Stallmann, Stefanie Schmitt, Julius Mezger, Christoph Bode, Daniel Duerschmied

**Affiliations:** 1 Department of Cardiology and Angiolog**y** I, Heart Center, University of Freiburg, Freiburg, Germany; 2 Faculty of Biology, University of Freiburg, Freiburg, Germany; INSERM, France

## Abstract

**Objective:**

Activated platelets release serotonin at sites of inflammation where it acts as inflammatory mediator and enhances recruitment of neutrophils. Chronic treatment with selective serotonin reuptake inhibitors (SSRI) depletes the serotonin storage pool in platelets, leading to reduced leukocyte recruitment in murine experiments. Here, we examined the direct and acute effects of SSRI on leukocyte recruitment in murine peritonitis.

**Methods:**

C57Bl/6 and Tph1−/− (Tryptophan hydroxylase1) mice underwent acute treatment with the SSRI fluoxetine or vehicle. Serotonin concentrations were measured by ELISA. Leukocyte rolling and adhesion on endothelium was analyzed by intravital microscopy in mesentery venules with and without lipopolysaccharide challenge. Leukocyte extravasation in sterile peritonitis was measured by flow cytometry of abdominal lavage fluid.

**Results:**

Plasma serotonin levels were elevated 2 hours after fluoxetine treatment (0.70±0.1 µg/ml versus 0.27±0.1, p = 0.03, n = 14), while serum serotonin did not change. Without further stimulation, acute fluoxetine treatment increased the number of rolling leukocytes (63±8 versus 165±17/0.04 mm^2^min^−1^) and decreased their velocity (61±6 versus 28±1 µm/s, both p<0.0001, n = 10). In Tph1−/− mice leukocyte rolling was not significantly influenced by acute fluoxetine treatment. Stimulation with lipopolysaccharide decreased rolling velocity and induced leukocyte adhesion, which was enhanced after fluoxetine pretreatment (27±3 versus 36±2/0.04 mm^2^, p = 0.008, n = 10). Leukocyte extravasation in sterile peritonitis, however, was not affected by acute fluoxetine treatment.

**Conclusions:**

Acute fluoxetine treatment increased plasma serotonin concentrations and promoted leukocyte-endothelial interactions *in-vivo*, suggesting that serotonin is a promoter of acute inflammation. E-selectin was upregulated on endothelial cells in the presence of serotonin, possibly explaining the observed increase in leukocyte-endothelial interactions. However transmigration of neutrophils in sterile peritonitis was not affected by higher serotonin concentrations, indicating that the effect of fluoxetine was restricted to early steps in the leukocyte recruitment. Whether SSRI use in humans alters leukocyte recruitment remains to be investigated.

## Introduction

The majority of peripheral (i.e. non-neuronal) serotonin is produced by entochromaffin cells in the mesentery and stored in the dense granules of platelets [1], [2]. The mechanisms of platelet uptake, storage, and targeted release are similar to those in neurons except for the fact that platelets circulate throughout the vasculature and are not stationary. Several different effects of peripheral serotonin have been unraveled in the past, including prohemostatic [3], [4], mitogenic [5], [6], and immunomodulatory [7], [8], [9] functions. We recently found, that platelet serotonin is an important mediator in the recruitment of neutrophils to sites of acute inflammation [10].

Platelets are not capable of synthesizing serotonin with the consequence that the platelet serotonin storage pool can be depleted by pharmacological blockade of the uptake mechanism via the serotonin transporter (SERT) with selective serotonin reuptake inhibitors (SSRI) [10]. Fluoxetine, a standard SSRI, influences plasma and serum serotonin concentrations after acute and chronic treatment in animal models and in patients with clinical depression [11], [12], [13], [14], [15]. While the depletion of platelet serotonin pools by chronic fluoxetine treatment is very well established, the acute effects of fluoxetine on plasma, i.e. not platelet-influenced, and serum, i.e. in large parts platelet-derived, serotonin is not well examined. Especially harsh plasma preparation techniques without diligent platelet silencing rapidly induce platelet activation with the release of highly concentrated serotonin. Acute treatment with fluoxetine inhibited tumor necrosis factor-α-release from monocytes into serum in lipopolysaccharide-challenged mice, suggesting immunomodulatory effects [16].

This lead us to investigate the effects of acute fluoxetine treatment on serum and plasma serotonin levels, leukocyte-endothelial interactions, and leukocyte extravasation with respect to whether this was serotonin-mediated or rather attributed to direct fluoxetine effects on endothelium, platelets, or leukocytes. We studied wild type and Tph1−/− mice, which are genetically deficient of non-neuronal serotonin [17]. Tryptophan hydroxylase (Tph) 1 is a rate-limiting enzyme mediating the synthesis of serotonin in enterochromaffin cells (primary source), pulmonary endothelial cells, mast cells, and monocytes/macrophages [17], [18], [19], [20]. The neuronal isoform Tph2 is not affected in these mice [17]. Platelets highly efficiently take up enterochromaffin cell-derived serotonin from plasma via SERT [3]. We show that acute fluoxetine treatment increases plasma serotonin levels and modulates leukocyte-endothelial interactions by increasing the surface expression of E-selectin on endothelium.

## Materials and Methods

### Mice

C57BL/6 mice were purchased from Charles River (Sulzfeld, Germany). Tph1−/− mice were kindly provided by M. Bader, Max-Delbruck-Center, Berlin, Germany and were on C57BL/6J background [17]. All animal experiments were performed in compliance with the German animal protection law (TierSchG). The mice were housed and handled in accordance with good animal practice as defined by FELASA (www.felasa.eu/guidelines.php) and the national animal welfare body GV-SOLAS (www.gv-solas.de/index.html). The animal welfare committee of the University of Freiburg as well as the local authorities (Regierungspräsidium Freiburg) approved all animal experiments.

### Acute Fluoxetine Treatment

To block the uptake of plasma serotonin into platelets, 6 week-old C57BL/6 or Tph1−/− mice were injected intraperitoneally with fluoxetine (40 mg/kg; Sigma-Aldrich) two hours prior to evaluation. Control mice received the same amount vehicle i.p.

### Quantification of Serotonin in Serum and Plasma

To estimate the biologically available serotonin content in whole blood, serum was prepared by strong single-agonist activation (thrombin) of platelets and coagulation. Blood was collected from the retro-orbital sinus with non-coated glass capillaries and incubated with 0.1 U/mL thrombin for 30 minutes at room temperature (Sigma-Aldrich). Clot and blood cells were pelleted by centrifugation at 1,000 g for 5 minutes and serum was purified by centrifugation at 16,000 g for 5 minutes. Plasma was prepared by retro-orbital blood collection with heparin-coated glass capillaries in 10 mM EDTA (Gerbu), followed by pelleting of blood cells by centrifugation at 1,000 g for 5 minutes and purification by centrifugation at 16,000 g for 1 minute. Serotonin concentration was measured with the Serotonin Fast Track ELISA (Labordiagnostika Nord, Germany) according to the manufacturer’s instructions.

### Platelet-rich Plasma Stimulation with Fluoxetine

Citrated blood was collected from the retro-orbital sinus of 8 week-old WT mice and centrifuged at 100 g for 10 minutes to obtain platelet-rich plasma (PRP). PRP was incubated with fluoxetine (30 and 60 mg/ml) or vehicle for 15 minutes at room temperature, followed by centrifugation at 1.000 g for 5 minutes and purification at 16.000 g for 1 minute. Serotonin concentration in the supernatant was then measured by ELISA (Labordiagnostika Nord, Germany).

### Intravital Microscopy of Mesenteric Veins

Six week-old male mice were pretreated with an intraperitoneal (i.p.) injection of 20 mg/kg E. coli serotype 055:B5 lipopolysaccharide (LPS) or vehicle 4 hours prior to the experiment. The mice also received a pretreatment with fluoxetine or vehicle 2 hours prior to the experiment. Mice were anesthetized with 100 mg/kg ketamine and 5 mg/kg xylazine i.p. Platelets and white blood cells were fluorescently labeled by retroorbital injection of 50 µl rhodamine 6G (1 mg/mL, Sigma-Aldrich). After median laparotomy a loop of ileum was exteriorized in a temperature-controlled, humidified plastic chamber, and a small mesenteric vein with a diameter of approximately 200 µm was visualized with an Axiovert 200 M inverted microscope and an AxioCam MRm camera using AxioVision Rel. 4.6 software (Zeiss). Blood cell interactions with the endothelium were recorded for 1 minute in 4 veins/mouse and averaged. Leukocyte velocity was determined by measuring the time one single leukocyte needed to pass a distance of 100 µm while stably rolling on endothelium. Leukocyte adhesion was defined as no visible movement for 30 seconds. All analyses were carried out off-line by an independent investigator blinded to genotype and treatment.

### Sterile Peritonitis and Flow Cytometry

Eight to twelve week-old male mice were treated with 1 ml of 4% thioglycollate i.p. for chemical irritation (0.6 mg/kg i.p., Sigma-Aldrich) or vehicle. After 4 hours cells were recovered by peritoneal lavage with 8 ml of PBS without Mg^2+^ and Ca^2+^ and counted by flow cytometry. For milder inflammatory conditions mice were treated with 200 µl of 4% thioglycollate and peritoneal lavage was performed after 3 hours. Cells were stained with an APC-labeled rabbit anti-mouse Gr-1 antibody (eBioscience, Germany) and read on a FACSCalibur flow cytometer (Becton Dickinson, Germany). The number of Gr-1 positive cells was counted in relation to a known number of added Sphero rainbow beads (Spherotech, USA) using the analyzing software FlowJo 7.6.3 (Tree Star, USA).

### Endothelial Stimulation with Serotonin

8 week-old WT and Tph1−/− mice were injected with serotonin (50 mg/kg) i.p. 2 hours before tissue harvest of mesenteric vessels.

### Tissue Harvesting and Histology

For histological analysis of mesenteric veins, the mesenteries were exteriorized and the intestines were cautiously removed. The tissue was embedded in OCT Tissue Tec (Sakura Finetec, Netherlands) and frozen for histology. Frozen tissue was cut in 10 µm sections. Immunofluorescence was performed to analyze the expression of E-Selectin on endothelial cells. A goat anti-mouse CD62E polyclonal antibody (Santa Cruz, USA) was used without permeabilization to stain surface E-selectin. The primary antibody was detected using a rabbit anti-goat CF 594 antibody (Sigma, Germany). Nuclei were stained by DAPI. E-selectin levels on mesenteric vessels were semi-quantified by measuring pixel intensity divided by vessel area using Image J (NIH).

### Statistical Analysis

Data are presented as mean ± SEM and were analyzed with GraphPad Prism (GraphPad Software, Inc., USA). Groups were compared by unpaired, two-tailed student *t* test or 1-way ANOVA followed by Bonferroni’s correction. *P* values <0.05 were regarded as statistically significant.

## Results

### Acute Treatment of WT Mice with Fluoxetine Slightly Elevated Plasma Serotonin Levels

**Figure 1:**
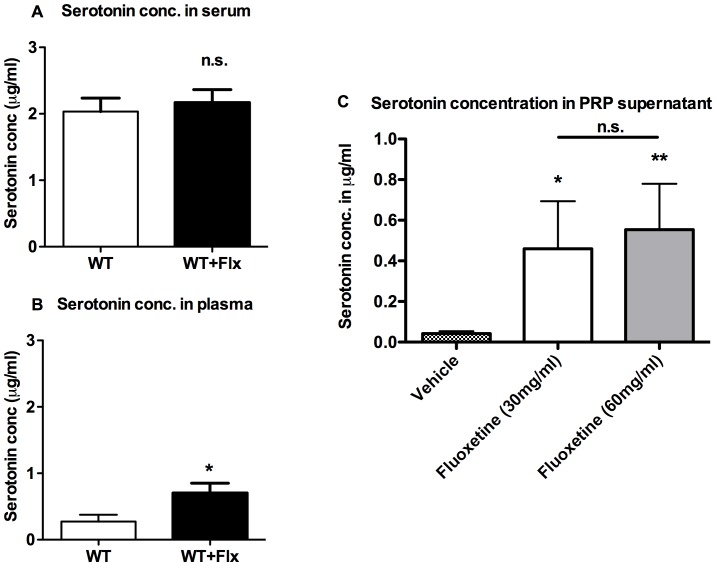
Serotonin levels in serum and plasma. Serotonin concentrations in serum (A), plasma (B) and supernatant of PRP after fluoxetine challenge (C) measured by ELISA (n = 14). n.s. = not significant, * = p<0.01.

To test how the blockade of SERT influences plasma and serum serotonin concentrations, fluoxetine was injected i.p. two hours prior to investigation. Serum serotonin levels did not change significantly (Figure 1A), suggesting that storage in platelets was not influenced during this short treatment period. Plasma serotonin levels were significantly increased in fluoxetine-treated mice (0.3±0.1 vs. 0.7±0.1 µg/ml, n = 14; p = 0.03; Figure 1B), suggesting rapid but low-level accumulation in plasma. Serotonin levels where also significantly higher in the supernatant of PRP that was incubated with fluoxetine, suggesting a high turnover of serotonin in platelets blocked by fluoxetine (Figure 1C).

### Leukocyte-endothelial Interactions Increased after Acute Fluoxetine Treatment when Peripheral Serotonin was Present

**Figure 2:**
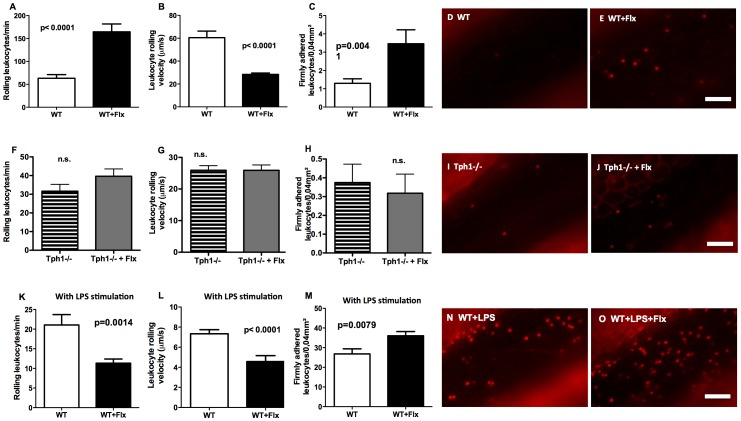
Leukocyte-endothelial interactions were amplified by acute fluoxetine treatment without further stimulation. Rhodamine-stained leukocyte rolling (A), velocity (B) and adhesion (C) in resting mesenteric veins and representative intravitalmicroscopy images of vehicle- (D) and fluoxetine-treated (E) WT mice (n = 10). Leukocyte rolling (F), velocity (G) and adhesion (H) in resting mesenteric veins and representative intravitalmicroscopy images of vehicle- (I) and fluoxetine-treated (J) Tph1−/− mice (n = 10). Rhodamine-stained leukocyte rolling (K), velocity (L) and adhesion (M) in lipopolysaccharide stimulated mesenteric veins 4 hours prior to investigation and representative intravitalmicroscopy images of vehicle- (N) and fluoxetine-treated (O) WT mice (n = 10). n.s. = not significant. Flx = fluoxetine.

Leukocyte-endothelial interactions were examined using intravital microscopy. Two hours after fluoxetine treatment and without further stimulation of mesenteric veins the number of rolling leukocytes was significantly elevated compared to vehicle injection (63.3±7.9 vs. 164.6±17 rolling leukocytes/min, n = 10; p<0.0001; Figure 2A). The velocity of rolling leukocytes was significantly decreased after fluoxetine administration (28.4±1.1 vs. 60.5±5.7 µm/s, n = 10; p<0.0001; Figure 2B). Firm adhesion was slightly increased after fluoxetine treatment (1.3±0.2 vs. 3.5±0.8 firmly adherent leukocytes/0.04.mm^2^, n = 10; p = 0.0041; Figure 2C). In the absence of peripheral serotonin in Tph1−/− mice, leukocyte rolling, velocity, and adhesion were not significantly influenced by fluoxetine treatment (Figure 2F–H).

### Leukocyte-endothelial Interactions in LPS-induced Inflammation were Enhanced by Acute Fluoxetine Treatment

Mesenteric vessels were stimulated in WT mice by LPS injection prior to the experiment. As expected, this reduced the number of rolling leukocytes, decreased their velocity, but increased firm adhesion 25-fold (Figures 2K–O). Leukocyte rolling and especially rolling velocity was further decreased by acute fluoxetine treatment (4.6±0.6 vs. 7.3±0.4 µm/s, n = 10; p<0.0001). Firm leukocyte adhesion however was increased after fluoxetine treatment compared to LPS stimulation alone (36±2.1 vs. 26.8±2.6 leukocytes/0.04 mm^2^, n = 10; p = 0.0079).

### Serotonin Accumulation after Acute Fluoxetine Treatment Increased the Surface Expression of E-selectin in Venous Endothelium

**Figure 3:**
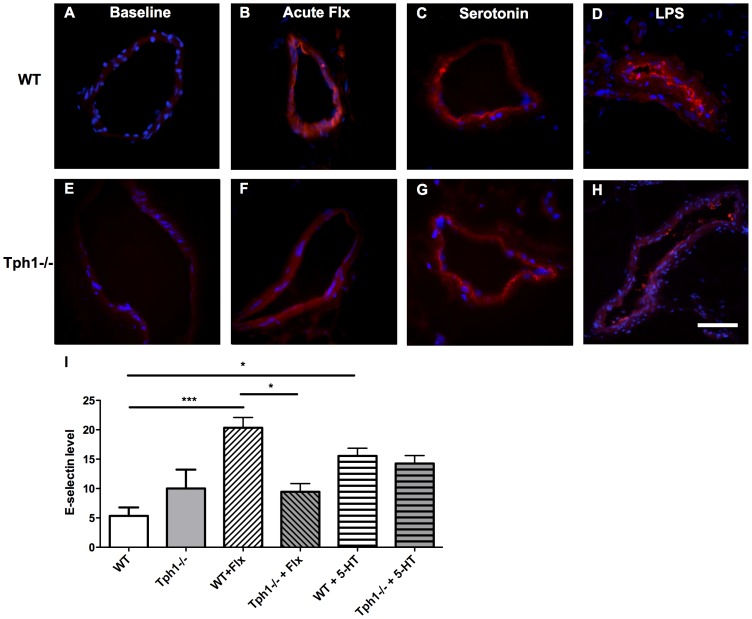
E-selectin expression is upregulated in the presence of peripheral serotonin. Immunofluorescence for E-selectin (red) and nuclei (blue). Mesenteric venules at baseline conditions (A), after fluoxetine treatment (B), after serotonin challenge (C) and after lipopolysaccharide stimulation (D) of WT mice. Mesenteric venules of Tph1−/− mice at baseline (E), after fluoxetine treatment (F), after serotonin challenge (G) and after lipopolysaccharide stimulation (H). Semi quantification of fluorescence-levels for E-selectin (I). Scale bar = 50 µm; Flx = fluoxetine, LPS = lipopolysaccharide. ** = p<0.001.

Immunofluorescence was used to investigate the surface expression of the adhesion receptor E-selectin on endothelium. In WT and Tph1−/− mice without specific treatment, only negligible signal for E-selectin appeared (Figures 3A,E). Acute fluoxetine treatment induced distinct but discontinuous surface E-selectin staining in WT mice, while this signal was weaker in Tph1−/− mice (Figures 3B,F). Serotonin infusion induced discontinuous surface E-selectin expression in both WT and Tph1−/− mice (Figure 3C,G). After stimulation with LPS a strong E-selectin signal delineated on the endothelial surface in WT but not in Tph1−/− mice, where the signal was weaker and not as continuously circumferential (Figures 3D,H). Semi-quantification of E-selectin levels on venules confirmed significantly higher levels after acute fluoxetine treatment compared to vehicle in WT mice (20.3±1.8 vs. 5.2±1.5, n = 4–6; p<0.005; Figure 3I).

### Neutrophil Extravasation in Sterile Peritonitis was not Influenced by Acute Fluoxetine Treatment

**Figure 4:**
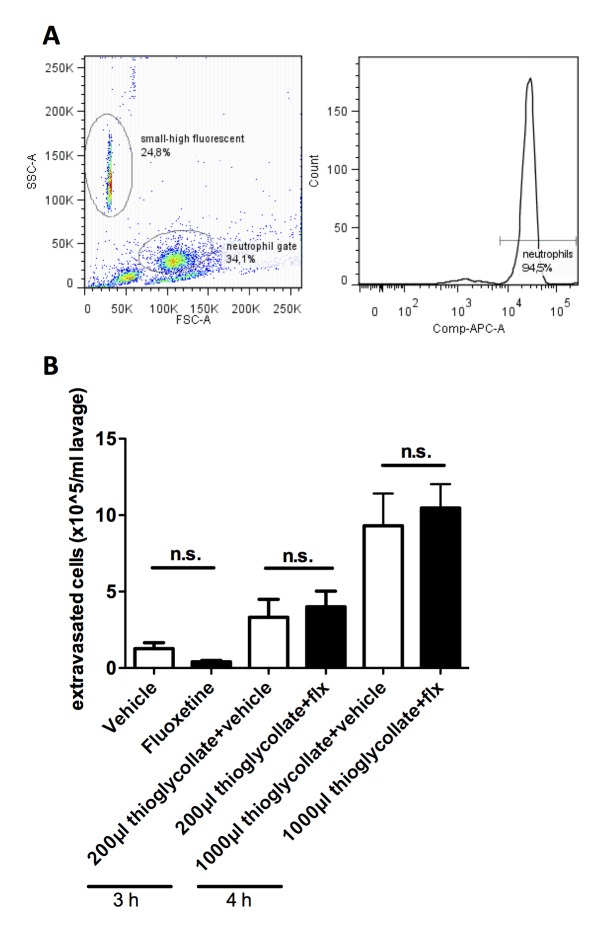
Neutrophil extravasation is unaltered after acute fluoxetine treatment in vivo. Gating strategies for Gr-1 neutrophil count with flow cytometry (A). Number of Gr-1 positive neutrophils in abdominal lavages of vehicle- and fluoxetine-treated WT mice 4 hours after intraperitoneal injection of 4% thioglycollate or vehicle (B, n = 9–10). n.s. = not significant.

We finally measured the influence of acute fluoxetine treatment on neutrophil transmigration into the peritoneal cave during thioglycollate peritonitis. Acute fluoxetine treatment alone did not induce neutrophil extravasation (1.3±0.4 vs. 0.4±0.1×10^5^/ml, n = 9; Figure 4B). Moreover, acute fluoxetine treatment did not significantly influence mild (3,32±1,2 vs. 4±1×10^5^/ml, n = 5) and strong thioglycollate-induced extravasation (9.3±2.1 vs. 10.5±1.6×10^5^/ml, n = 10).

## Discussion

In mice, the acute administration of SSRIs has anti-inflammatory effects and hence improves survival of septic shock, at least in part by modulating the release of cytokines [16]. We have previously found that the chronic treatment of mice with the standard SSRI fluoxetine attenuates neutrophil adhesion and rolling on endothelial cells, possibly also mediating protection from septic shock [10]. This effect was very similar to the phenotype seen in Tph1−/− mice. It was attributed to the depletion of intracellular serotonin stores in circulating platelets by continuous administration of fluoxetine for 21 days. We concluded that platelet serotonin promotes the recruitment of neutrophils to sites of acute inflammation. We also confirmed that platelet serotonin is a proinflammatory mediator in allergic airway inflammation, promoting peribronchiolar inflammatory cell recruitment [21]. Again, Tph1−/− mice were protected from an inflammatory disorder, in this case the autoimmune disease asthma. Acute treatment with SSRIs decreases the degree of bronchial hyperreactivity in mice and affects inflammatory signaling in vitro and in vivo [16], [22], but it is unclear if - and how - the acute administration of SSRI affects leukocyte-endothelial interactions. We present murine in vivo data elucidating the immediate consequences of fluoxetine administration to fill this gap in knowledge about a drug that many patients suffering from depression take on a regular basis. Soon after these patients start taking an SSRI for the first time, their plasma serotonin levels likely increase in a similar way as our murine data indicate. Our results may hence be relevant for depressed patients, if future clinical studies confirm these findings in humans.

Here, we describe a surprising increase in leukocyte rolling on otherwise unstimulated endothelium of mesenteric veins by more than 150% two hours after administration of fluoxetine into the abdominal cavity as compared to administration of vehicle alone. The injection of fluoxetine also more than doubled the number of firmly adhered leukocytes. In comparison to earlier studies without intraperitoneal injection, more leukocyte adhesion was observed, even without fluoxetine [10], [23], [24]. Most likely, this difference can be explained by endothelial stimulation induced by mechanical irritation provoked by the process of injecting a liquid into the abdominal cavity that cannot be avoided. This prestimulation however was equal in all groups and controlled for by vehicle administration in the same way as fluoxetine administration. Further challenge by LPS still induced a more than 25-fold increase in leukocyte adhesion, confirming that full endothelial activation was still possible.

Interestingly, acute fluoxetine administration enhanced LPS-induced adhesion by 38%. At the same time, the rolling velocity had decreased, because the rolling leukocytes had slowed down significantly under the influence of fluoxetine. These observations are highly suggestive for a relevant upregulation of endothelial adhesion receptors. While several of these receptors could have been influenced, we decided to focus on E-selectin in this study, because E-selectin is responsible for the deceleration and slow rolling [25], [26], [27]. Indeed, fluoxetine and the infusion of serotonin induced an upregulation of endothelial surface E-selectin. Investigating the other adhesion receptors, such as P-selectin, vascular cell adhesion molecule-1 (VCAM-1), or intercellular adhesion molecule-1 (ICAM-1) may be an interesting part of future studies.

We next sought to address the mechanism mediating the acute fluoxetine effect. After acute fluoxetine treatment the content of serotonin in dense granules of platelets remained constant, but serotonin levels in plasma more than doubled. Incubation of PRP with fluoxetine mimicked this effect in vitro. This was likely caused by an unaffected serotonin synthesis in enterochromaffin cells and a high turnover of serotonin in platelets, while uptake to platelet dense granules via SERT had already been blocked [28]. A balance between synthesis and oxidation by monoaminooxidases and renal elimination will be reached at some point, but our data do not allow any conclusions about the levels that will be reached in plasma, because we did not perform time-course experiments. However, our results show an association between the acute increase in plasma serotonin levels after fluoxetine administration and endothelial E-selectin upregulation several hours later that suggests that serotonin may induce the presentation of E-selection on the endothelial surface. While other effects and in particular a direct action of fluoxetine on endothelium cannot yet be excluded, these findings are in line with the results of earlier studies. Serotonin has been described to induce Weibel-Palade body release, to induce tissue factor and plasminogen activator inhibitor-1 expression, and to influence the expression of ICAM-1 and VCAM-1 following inflammatory stimulation on cultured endothelium [10], [29], [30], [31]. Therefore, we propose increased - slow - leukocyte rolling on E-selectin as a possible consequence of fluoxetine administration due to accumulation of free serotonin in the plasma. This conclusion was strengthened by the observation, that serotonin infusion elevated E-selectin expression both in WT and Tph1−/− mice, while fluoxetine treatment did not elevate E-selectin expression in Tph1−/− mice.

While LPS-induced leukocyte adhesion was enhanced, leukocyte extravasation into the abdominal cavity following thioglycollate challenge was not affected by fluoxetine. The most likely explanation for this seemingly contradictory finding is a relatively weak effect of acute fluoxetine treatment on leukocyte recruitment: The (probably plasma serotonin-mediated) endothelial activation after acute fluoxetine treatment may be overcome by the strong unspecific activation induced by the chemical irritant thioglycollate. Significant serotonin effects in the setting of strong, acute inflammation most likely depend on the targeted release of serotonin from activated platelets at high concentrations rather than lower plasma levels [10]. Accordingly, Tph1−/− mice display reduced neutrophil extravasation in thioglycollate-induced peritonitis [10].

In line with these arguments, there was no influence of acute fluoxetine treatment on leukocyte-endothelial interactions in Tph1−/− mice, because peripheral serotonin was absent [17]. This strengthens the proposed mechanism of plasma serotonin mediating the effects of acute fluoxetine administration. Hence, in summary, we present a novel pro-inflammatory effect of acute treatment with SSRIs. The injection of fluoxetine promoted slow leukocyte rolling on the endothelium of mesenteric venules, which most likely mediated by a plasma serotonin-induced increase in E-selectin expression.
