# Correction: Acute Fluoxetine Treatment Induces Slow Rolling of Leukocytes on Endothelium in Mice

**DOI:** 10.1371/journal.pone.0142762

**Published:** 2015-11-06

**Authors:** Nadine Herr, Maximilian Mauler, Thilo Witsch, Daniela Stallmann, Stefanie Schmitt, Julius Mezger, Christoph Bode, Daniel Duerschmied

The following funder is missing from the Funding section: Deutsche Herzstiftung. The correct funding information is as follows: This work was funded by the Deutsche Forschungsgemeinschaft (DFG) and the Deutsche Herzstiftung. The funders had no role in study design, data collection and analysis, decision to publish, or preparation of the manuscript.
